# Partial herd hoof trimming results in a higher economic net benefit than whole herd hoof trimming in dairy herds

**DOI:** 10.1371/journal.pone.0301457

**Published:** 2024-04-02

**Authors:** Marlena Waldbauer, Eldon Spackman, Herman W. Barkema, Edmond A. Pajor, Sebastian Knauss, Karin Orsel

**Affiliations:** 1 Department of Production Animal Health, Faculty of Veterinary Medicine, University of Calgary, Calgary, AB, Canada; 2 Department of Community Health Sciences, Cumming School of Medicine, University of Calgary, Calgary, AB, Canada; 3 Casper Trimming Ltd., Olds, AB, Canada; Eskisehir Osmangazi University: Eskisehir Osmangazi Universitesi, TURKEY

## Abstract

Sole ulcers, a common cause of lameness is the costliest non-infectious foot lesion in dairy cows and one of the most prevalent non-infectious foot lesions in freestall housing systems. Costs associated with sole ulcers are treatment costs, plus increased labor and decreased productivity and fertility. Routine hoof trimming is part of a strategy to manage sole ulcers. However, hoof trimming strategies differ among farms. The two most frequently applied strategies are: 1) partial herd hoof trimming with a 2-month interval between trims; and 2) whole herd hoof trimming at 6-month intervals. A Markov model was developed to investigate whether every 2 months partial herd hoof trimming or whole herd hoof trimming every 6 months was the most cost-effective strategy to avoid costs associated with sole ulcers. In this model, the net benefits for a 100-cow herd and the average productive life span of a dairy cow in intensive dairy systems of 3 years were evaluated. Partial herd hoof trimming was the most cost-effective strategy 100% of the time compared to whole herd hoof trimming, with a difference in 3-year net benefits of US$4,337 (95% CI: US$2,713—US$5,830). Based on sensitivity analyses, variables that were the sources of the biggest uncertainty in the model were herd size, the probability of being trimmed in a partial herd trim, and the prevalence of sole ulcers. To further investigate the impacts of herd size and of probability of being trimmed, various scenario analyses were conducted. With increasing herd size, the difference in net benefits in favor of partial herd hoof trimming increased even more. Scenario analyses about the probability of getting trimmed all indicated that targeted intervention increased the difference in net benefits in favor of partial herd hoof trimming. However, if the selection of cows to be trimmed in a partial herd trim was random, the whole herd hoof trimming strategy became cost-effective. Therefore, targeted selection and early intervention are necessary to decrease costs associated with sole ulcers.

## Introduction

Lameness is most often caused by an infectious or non-infectious lesion of the foot [1]. Sole ulcers (SU) are non-infectious foot lesions; they are described in the International Committee for Animal Recording (ICAR) Claw Health Atlas as “Penetration through the sole horn exposing fresh or necrotic corium” [2] and they resulted in lameness in 54% of lesion presence [3]. The pathophysiology of SU is complex and in parts unclear, but includes environmental risk factors, physiological events in the cow’s life cycle, hoof overgrowth, and external factors, e.g., trauma/changes inside the hoof capsule [4], with SU visible on the surface within 4 to 6 weeks after trauma but potentially even longer [5]. Changes in locomotion are possible before the lesion is visible. The time needed for a new layer of horn to cover an SU is described to be four weeks for uncomplicated cases [6]. Cows with SU were no longer lame in 75% of the cases after 35 days when properly treated [7]. In another study, 85% of cows with SU recovered between the two trimmings performed in autumn and in spring [8]. SU appeared to only be weakly associated with genetic susceptibility [9].

In Europe and the United States, the reported prevalence of SU ranged from 3 to 28% [10, 11]. For example, in the Netherlands, SU were the most prevalent foot lesion, affecting 5.6% of cows enrolled in the study and in 85% of study herds [12]. Furthermore, SU were the most common non-infectious foot lesion in Alberta dairy cows, with a prevalence of 6.4% in cows presented to a hoof trimmer [13], as well as in Ontario, with a 12.5% herd-level prevalence and 89.5% of herds affected [14].

SU are an animal welfare issue as they can cause pain, and potentially alter gait [15, 16]. Therefore, there is a moral obligation to treat cows affected by SU promptly. However, a New Zealand study indicates that improvement in shortening the time between detection and treatment is needed [17]. An Alberta study also suggests that there is still an issue in recognizing lame cows that need intervention [18].

SU are associated with economic losses [19–21]. In addition to costs for treatment, decreased productivity [22] and fertility [23], also the correlation with mastitis [24] need to be considered. Moreover, cows with SU increase labor costs because cows need special attention (e.g., moving cows to another barn, cows needing more time to enter the parlor, and sorting cows for the hoof trimmer). With estimated costs per case of SU of US$216, SU are reported to be the most expensive non-infectious hoof lesion, with the most recent estimation of milk loss being the main contributor to the costs of SU [20]. However, we also know that not all cows diagnosed with SU have concurrent gait abnormalities or producers are unsuccessful in identifying lame cows [3, 18].

Hoof trimming is a management strategy with both, preventive and therapeutic potential, for SU [25, 26], with regular hoof trimming commonly recommended [8]. Two different hoof trimming strategies as a management practice in Alberta, Canada, were reported: 1. partial herd hoof trimming, where approximately 18% (±18.3) of the herd is trimmed every 2 months; 2. whole herd hoof trimming, with 6-month intervals between trims [13].

For an increased insight into the economic consequences, Markov models can be used as they enable the simulation of a cohort moving through various health states, modeling the costs and benefits of comparative interventions that account for risks of recurring events over a long-term time horizon [27].

The objective of this study was to compare the costs and benefits associated with partial herd hoof trimming every 2 months and whole herd hoof trimming every 6 months for the management of SU in dairy cows. To evaluate these effects, we used a Markov model over a 3-year time horizon and applied it to a cohort of dairy cows, with and without SU, that were exposed to these two common hoof trimming strategies over the lifespan of an average dairy cow [28].

## Material and methods

### Data sources

Inputs for the Markov model were obtained through a literature search in PubMed [29] and Google Scholar [30]. Model inputs not identified in the literature were generated from databases from previous lameness research [3, 13], industry sources, or expert opinion. Expert opinion was generated from close collaboration between veterinarians with extensive experience in lameness management in dairy herds (the authors MW, HWB, and KO) as well as hoof trimmers (the author SK as member of the Western Canadian Certified Hoof Trimmer’s Association (WCCHTA) presented data sources based on extensive field experience throughout the entire process). In the source section of the tables, it is referred to expert opinion where applicable. Model inputs are presented in Tables 1 and 2.

**Table 1:** Model inputs: Cohort (herd) size, prevalence, and probabilities, including calculations and sources.

Model inputs	Deterministic	Calculation	Probabilistic	Source
Cohort (herd) size	100			Expert opinion
Prevalence of sole ulcer	0.064		= BETA.INV(RAND(),1830,26777)	[13]
Probability of getting trimmed in partial trim—Overall	0.18		= BETA.INV(RAND(),43.74,199.26)	[13]
Probability of getting trimmed in partial trim–After trim no sole ulcer	0.1699		= BETA.INV(RAND(),0.1699*100,(100–0.1699*100))	Calculation
Probability of getting trimmed in partial trim–After trim sole ulcer	0.54		= BETA.INV(RAND(),853.7,727.3)	[3]
Probability of getting trimmed in partial trim–After no trim no sole ulcer	0.1699		= BETA.INV(RAND(),0.1699*100,(100–0.1699*100))	Calculation
Probability of getting trimmed in partial trim–After no trim sole ulcer	0.54		= BETA.INV(RAND(),853.7,727.3)	[3]
Cure probability of sole ulcer after trim	0.85		= BETA.INV(RAND(),2927.4,516.6)	[8]
Cure probability of sole ulcer without trimming	0.20		= BETA.INV(RAND(),0.20*100,(100–0.20*100))	Expert opinion
2-month probability of developing a sole ulcer	0.013	= 1-EXP(-(0.081*1/6))	^+^Calculated based on probabilistic value of incidence risk of sole ulcer	*Calculation based on incidence risk/incidence rate
*Incidence risk of sole ulcer	7.80%		^+^ = BETA.INV(RAND(),465,1624.7)	[31]
*Incidence rate	0.081	= -LN(1–7.80%)/1	^+^Calculated based on probabilistic value of incidence risk of sole ulcer	*Calculation based on incidence risk

**Table 2:** Model inputs: Benefits and costs, including *calculation of benefits and costs and sources.

Benefits and costs	Deterministic	Probabilistic	Source
Milk sales per cow per year	US$6,320.50	= NORM.INV(RAND(),8,208.44,0.1)	[32]
Net cattle sales per cow per year	US$284.60	= NORM.INV(RAND(),369.60,0.1)	[32]
1 Trim	US$15.02	= NORM.INV(RAND(),19.50,0.1)	Industry source–price in Alberta 2019
1 Block	US$20.25	= NORM.INV(RAND(),2.63,0.1)	*Calculation
Costs of ^a^AI without ^b^SU per cow	US$132.48	= NORM.INV(RAND(),172.05,0.1)	*Calculation
Costs of ^a^AI with ^b^SU per cow	US$220.80	= NORM.INV(RAND(),886.75,0.1)	*Calculation
Labor costs per partial herd trim	US$13.34	= NORM.INV(RAND(),17.33,0.1)	*Calculation
Labor costs per whole herd trim	US$2.96	= NORM.INV(RAND(),3.85,0.1)	*Calculation
Cost of an additional cow for 1 cow with ^b^SU	US$101.63	= NORM.INV(RAND(),131.99,0.1)	*Calculation
Costs of milk loss for a 2-month interval	US$73.50	= NORM.INV(RAND(),95.46,0.1)	*Calculation
*Calculation of Benefits and Costs	**Input for calculation**	**Value for calculation**	**Source**
1 Block, a product of	Cost of 1 block	US$28.88	Industry source–price in Alberta 2019
	Probability of getting a block when trimmed and having ^b^SU	70%	Data set of hoof trimming records of a 2018 UCVM lameness study, Expert opinion
Labor costs per partial herd trim	Average labor cost per hour	US$17.79	[32]
	Labor duration per trim	45 minutes	Expert opinion
Labor costs per whole herd trim	Average labor cost per hour	US$17.79	[32]
	Labor duration per trim	10 minutes	Expert opinion
Costs of ^a^AI (including insemination and hormones) without ^b^SU per cow per year	Costs of 1 ^a^AI (adjusted for currency and inflation)	US$44.35	[33]
	Number of ^a^AIs	3	[23]
Costs of ^a^AI (including insemination and hormones) with ^b^SU per cow per year	Costs of 1 ^a^AI (adjusted for currency and inflation)	US$44.35	[33]
	Number of ^a^AIs	5	[23]
Costs of additional cow per cow with ^b^SU	Reported cost of raising a dairy additional cow (adjusted for inflation)	US$2,032.71	[34]
	Factor of a cow needed to fill milk quota with ^b^SU	1/20 cow or 5% of a cow	^+^Calculated based on milk loss and milk yield [22] [35]
Costs of milk loss for a 2-month interval	^+^Milk loss over a lactation period when having ^b^SU	574 kg	[22]
	^+^Milk yield	10,909 kg	[35]
	Costs of milk loss	US$62.93 per hL	[32]

^a^AI(s): Artificial insemination(s)

^b^SU: Sole ulcer

### Model overview and inputs

A Markov model was created to compare the costs and benefits between the 2-months interval of partial herd hoof trimming and the 6-month interval of whole herd hoof trimming according to the Guidelines for the Economic Evaluation of Health Technologies: Canada [36]. The Markov model was created for 36 months to represent the average productive life span of an Albertan dairy cow of 3 years [28], which was also reported to be the average productive life span of dairy cows in other intensive dairy systems [37]. Therefore, either the model included 18 partial herd trims every 2 months or six whole herd hoof trimmings every 6 months. At each 2-month interval, four possible outcomes were included: 1) trimmed, ulcer present; 2) trimmed, no ulcer present; 3) not trimmed, ulcer present; and 4) not trimmed, no ulcer present. The cohort (herd) size for this model was 100 cows.

Also included in the model was an SU prevalence of 6.4% [13]. This determined the starting prevalence of the cohort in the Markov model. The 2-month probability of developing an SU between trims was calculated from the annual SU incidence risk of 7.8% reported in the literature [31]. For whole herd hoof trimming, each cow had a 100% probability of being trimmed; this resulted in 600 cow trimmings over 3 years, across six hoof trimming sessions. For partial herd hoof trimming, each cow had an 18% probability of being trimmed [13]. Therefore, in a 100-cow herd, 18 cows were trimmed every 2 months. This resulted in a total of 324 cow trimmings over 3 years. However, within this 18% overall probability, a cow with an SU was assumed to have a 54% chance to get trimmed, due to the presentation of abnormal gait or lameness resulting in selection for trimming by the producer [3]. If the cow was included or excluded in the next partial herd trimming depended on the “probability of getting trimmed in partial trim”, where the four possible probabilities in our Markov model are presented in Table 1. How often an individual cow was included in a partial herd trim in this study was therefore determined by the probabilities used in the Markov model. Therefore, according to the probabilities an individual cow could be included from the range from all or none partial herd trimmings depending on the four possible probabilities presented in Table 1. The probability of getting trimmed in a partial herd trim was in general higher with SU present than with no SU present. Costs allotted for each 2-month cycle were summed for the model length of 36 months (3 years). Benefits were calculated for 3 years for 100 cows. Both benefits and costs were discounted at 1.5%, as recommended by the Guidelines for the Economic Evaluation of Health Technologies: Canada [36] to account for the present value of future cash flows. Benefits and costs are provided in 2019 US Dollars (USD/US$).

Costs were related to direct costs, including the hoof trim itself as well as a block as needed. Moreover, costs related to decreased productivity (milk loss resulting in the need for an additional cow to fill the allotted quota) and fertility (five artificial inseminations [AI] for a cow with SU, compared to three AIs for a cow without SU), and increased on-farm labor, whereas benefits were derived from milk sales and cattle sales. Costs were either acquired from the Dairy Cost Study 2019 [32] in 2019 Canadian Dollars [38] or, if given in another currency, adjusted with the Bank of Canada Currency Converter in 2019 Canadian Dollars (USD/US$) [39]. Because US Dollars are more commonly used than Canadian Dollars the results were converted to 2019 US Dollars (CAN$1 = US$0.77) [38, 39].

The Canadian dairy industry is based on supply management; each producer has a milk quota and tries to fill that quota with the least number of cows possible, to obtain the highest economic return. If a cow does not produce to its full capacity, which is reported as 10,909 kg annually for a Holstein in Canada in 2019 [35], additional cows are needed to meet the quota. Due to an expected 5% production loss of a cow with SU, there is a need to replace this production loss, which has associated economic costs of raising additional replacements. This has been accounted for in the model through costs for a replacement cow (Table 3).

**Table 3:** Inputs for sensitivity analysis.

Inputs for sensitivity analysis	Lower limit	Upper limit	Calculation	Source of the lower and upper limit
Cohort (herd) size	50 cows	500 cows		Expert opinion
Prevalence of ^b^SU	1.4%	28%		[10] [40]
Probability of getting trimmed (partial herd)	10%	30%		Expert opinion
Cure probability with trim	68%	97%		[8]
Cure probability without trim	10%	40%		Expert opinion
Incidence risk of ^b^SU to calculate 2-month probability of developing ^b^SU	0.90%	31.50%		[31]
Cost of 1 trim	US$13.48	US$16.56		Industry source—prices in Alberta 2019
Cost of 1 block	US$26.95	US$30.80	Multiplied with the probability of block = 70%	Industry source—prices in Alberta 2019
Probability of getting a block	50%	95%	Multiplied with the cost of a block = US$28.88	Expert opinion
Cost of labor partial herd trim/whole herd trim	US$13.29/US$2.96	US$13.39/US$2.97		[32]
Reported cost of an additional cow	US$1,507.38*0.05 = US$75.37	US$2,707.87*0.05 = US$135.40	Multiplied with the factor of an additional cow needed to fill quota of a cow with SU = 5%	[41]
Cost of ^a^AI	Cost of ^a^AI—US$38.50	Cost of ^a^AI + US$38.50		Expert opinion
Milk sales per cow per year	US$5,802.82	US$6,698.71		[32]
Net cattle sales per cow per year	US$248.49	US$333.54		[32]

^a^AI: Artificial insemination

^b^SU: Sole ulcer

### Difference in net benefits

The difference in net benefits was the outcome of interest, calculated as the difference of benefits minus costs for both strategies. The benefits in this study did not represent the producer’s income, but the animal-related benefits comprised of milk sales and net cattle sales [32]. These benefits were used to create a baseline where the costs could be subtracted, to calculate the difference in net benefits. All costs were related to the hoof trim, with or without block, increased on-farm labor, and decreased productivity/fertility of a cow with SU.

### Probabilistic analysis

A probabilistic simulation was conducted using 2000 iterations, with no further change in the stability of results compared to 1000 iterations. The mean and the 95% confidence interval (CI) of these 2000 iterations were the values of interest, as these accounted for distributions around the deterministic variables and captured uncertainty in model parameters. Inputs for probabilities were selected based on a beta distribution, where the number of events was represented by alpha and non-events by beta distribution (Table 1). Probabilistic inputs for benefits and costs were based on normal distributions because there was very little uncertainty around prices used for the simulation (Table 2).

### Deterministic one-way sensitivity analysis

A sensitivity analysis was conducted to identify potential variables that are source(s) of uncertainty in the model. To capture the effect of a single variable change on the model output, a one-way sensitivity analysis was conducted [42]. The model was run with upper and lower limits as presented in the literature, by industry sources, or expert opinion (Table 3).

### Scenario analyses

To investigate alternative outcomes of the Markov model when changing specific values, that seemed to drive the model according to the sensitivity analysis, the following three scenarios were evaluated:

#### Scenario 1

A random probability for being trimmed was included, despite the presence or absence of SU. The probability was 18% overall, as well as 18% for cows having an SU and 18% for cows not having an SU. The model with these inputs was also run with 2,000 iterations in the probabilistic simulation.

#### Scenario 2

Three strategies of the probability of getting trimmed in a partial herd hoof trimming were compared: 1) A random strategy, where not just the overall probability of getting trimmed was 18% but also the probability of getting trimmed for a cow with SU; 2) the reference case with an 18% overall probability of getting trimmed and a 54% probability of getting trimmed when having a SU; and 3) a 18% overall probability of getting trimmed and a 100% probability of getting trimmed when having a SU. To ensure a direct comparison, the number of cows trimmed in these partial herd hoof trimmings remained at 324 over 3 years for all three options.

#### Scenario 3

The difference in net benefits depending on herd size (50, 100, 150, 200, 250 and 300, 350, 400, 450, and 500 cows) was investigated.

#### Scenario 4

The fourth scenario was conducted to allow for a comparison with dairy systems that do not have a quota system like Canada: The cost of the additional cow needed to fill the quota was replaced by direct costs of the milk loss, to reflect no supply management system. Inputs for calculation are presented in Table 2.

## Results

### Difference in net benefits

In the probabilistic reference case, the average difference in net benefits between the two strategies was US$4,337 (95% CI: US$2,713–5,830) over 3 years for a 100-cow herd, with partial herd hoof trimming being the cost-effective strategy in all scenarios. The deterministic results did not change the preferred strategy.

For partial herd hoof trimming and whole herd hoof trimming strategies alike, the factor associated with the biggest costs was the increased cost of reproduction, resulting in additional AIs for cows with SU, whereas the lowest cost was labor (Table 4). However, the costs of reproduction and labor were both lower for whole herd hoof trimming (Table 4). The other costs were higher for whole herd trim (Table 4), with this strategy being more costly overall.

**Table 4:** Means and 95% confidence interval (CI) of all costs, benefits, and the difference in net benefits from the probabilistic reference case given in 2019 USD for 3 years for a 100-cow herd. Partial herd trim cost-effective strategy 100% of the time. Whole herd trim cost-effective strategy 0% of the time.

	Partial herd trim			Whole herd trim		
Costs/Benefits (USD)	Mean	Lower limit 95% CI	Upper limit 95% CI	Mean	Lower limit 95% CI	Upper limit 95% CI
Cost of trim	$5,284	$3,923	$6,837	$9,007	$8,916	$9,104
Cost of block	$437	$356	$535	$624	$571	$678
Cost of reproduction	$41,693	$41,518	$41,877	$41,088	$40,967	$41,210
Cost of labor	$240	$237	$243	$18	$17	$19
Cost of an additional cow	$12,904	$11,669	$14,220	$14,229	$12,808	$15,776
Total cost	$59,672	$57,534	$61,861	$64,009	$62,449	$65,703
Benefits	$1,952,388	$1,952,388	$1,952,388	$1,952,388	$1,952,388	$1,952,388
**Difference in net benefits**	**US$4,337 (95% CI: US$2,713–5,830)**					

### Deterministic one-way sensitivity analysis

**Fig 1:**
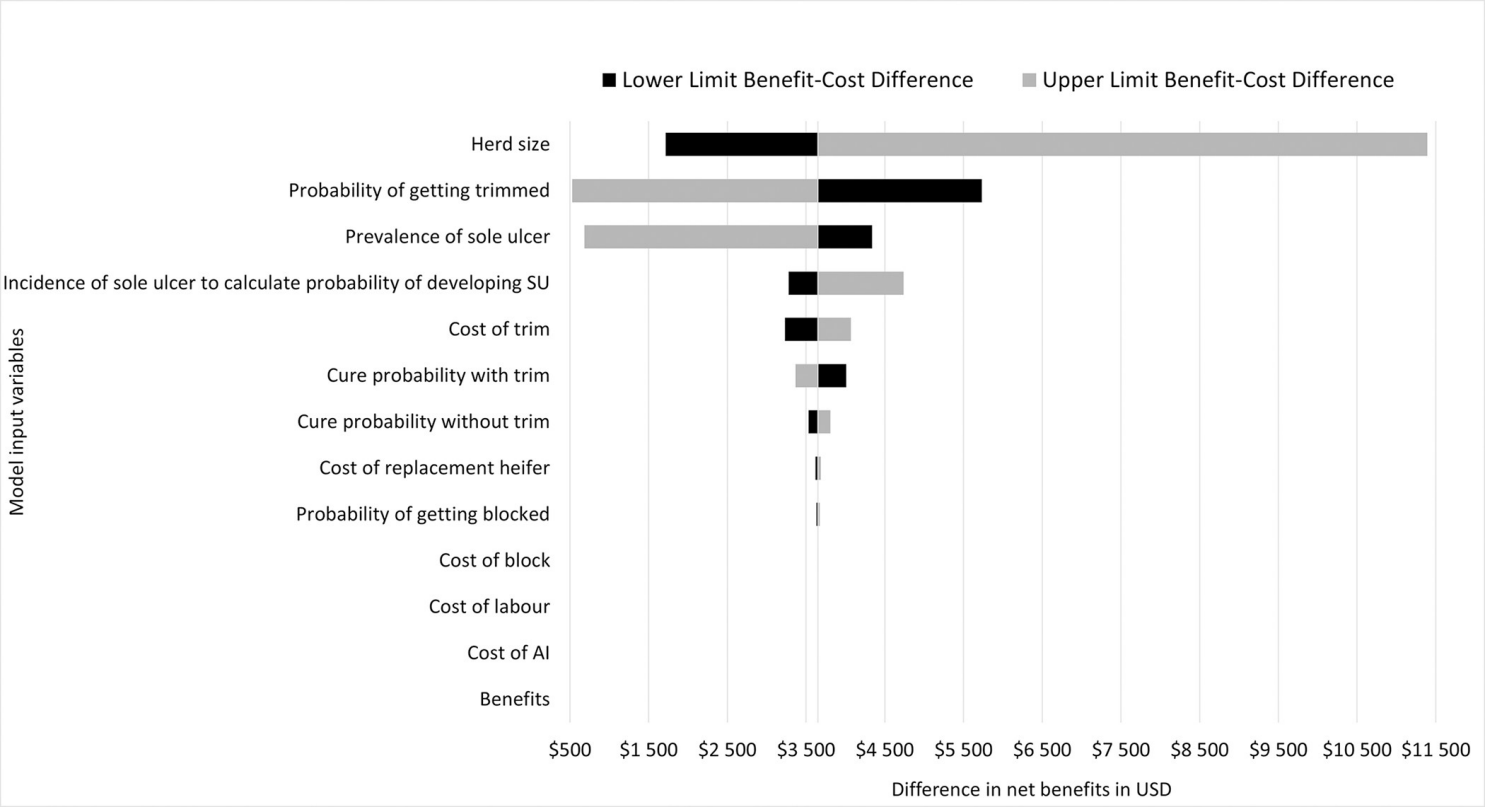
Tornado diagram of the deterministic one-way sensitivity analysis centered around the deterministic difference in net benefits of US$3,652.

None of the variables we varied in the model suggested a change in the cost-effective strategy from partial herd trim to whole herd trim. However, herd size, probability of being trimmed, and prevalence of SU resulted in the biggest differences in net benefits between partial herd hoof trimming and whole herd hoof trimming (Fig 1).

### Scenario analyses

#### Scenario 1

In the scenario where the probability of getting trimmed in the partial herd trim was random at 18%, not just overall but also for having an SU and not having a SU, whole herd hoof trimming was the most cost-effective strategy 98.6% of the time, with a difference in net benefits of US$3,041 (95% CI: US$344–6,409).

#### Scenario 2

**Fig 2:**
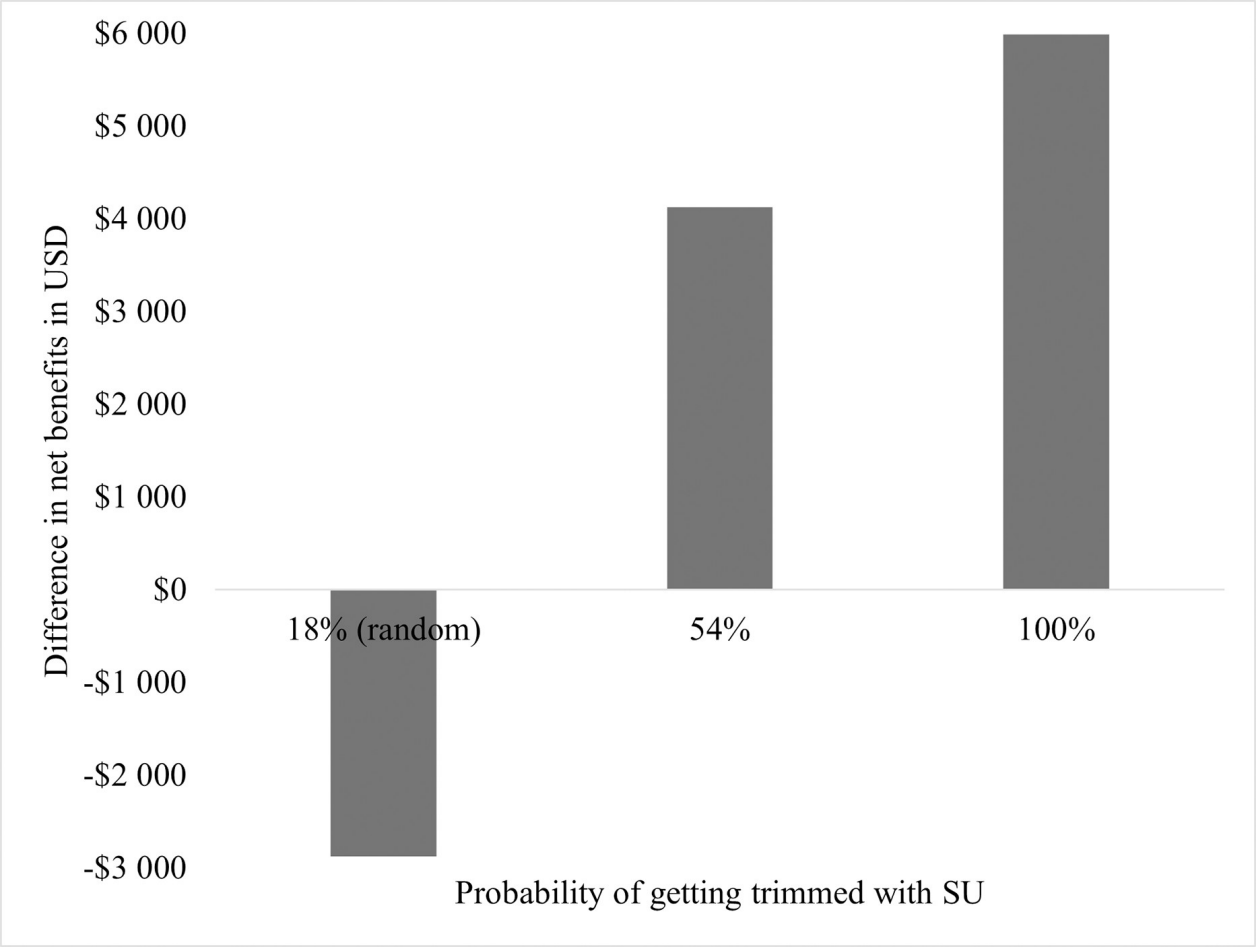
Mean difference in net benefits between partial herd hoof trimming and whole herd hoof trimming, depending on probability of getting trimmed.

In the comparison of the difference of net benefits, the net benefit of partial herd hoof trimming increased with the higher probability of identifying cows with SU, based on lameness, and therefore hoof trimming of these cows (Fig 2).

#### Scenario 3

**Fig 3:**
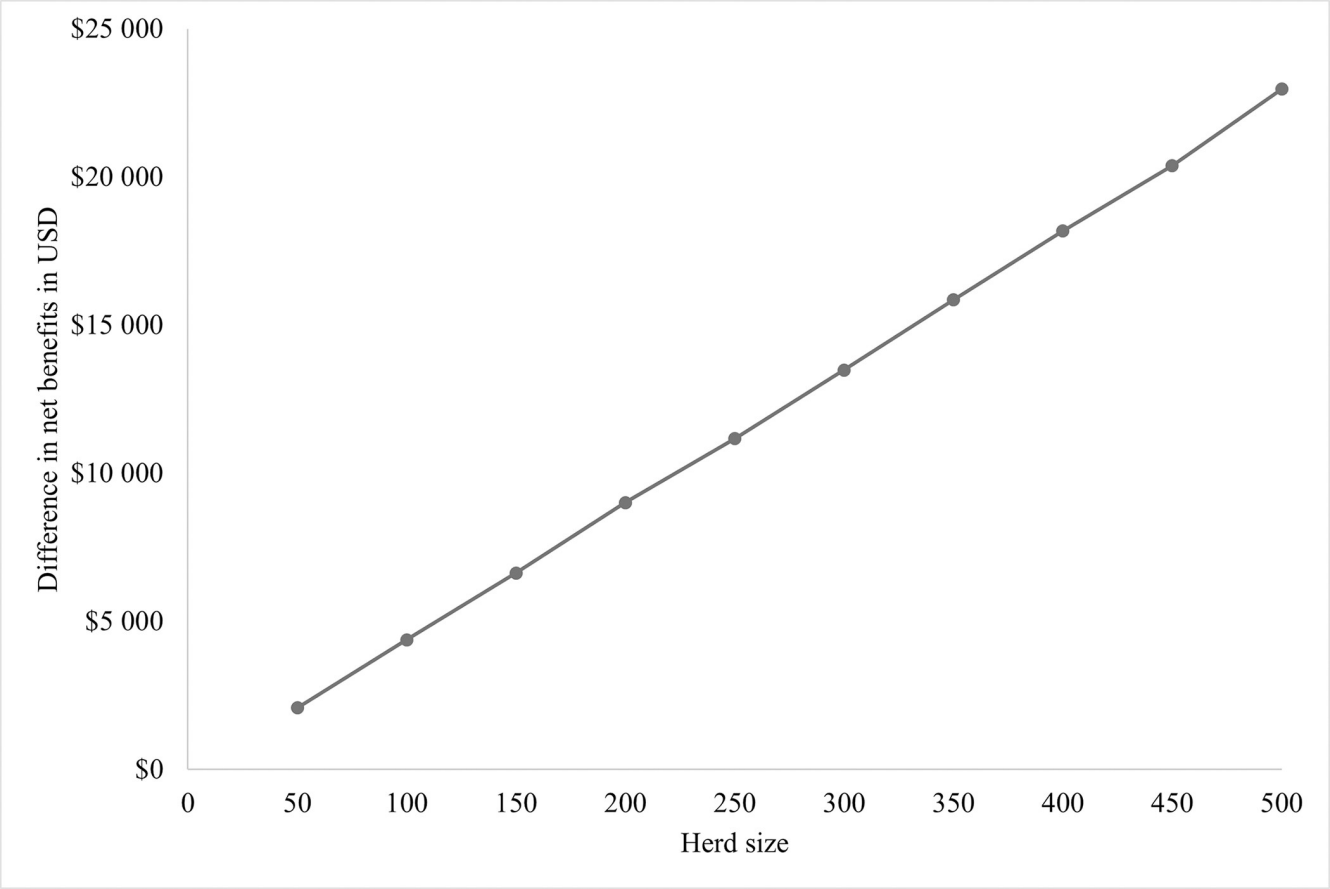
Mean difference in net benefits between partial herd hoof trimming and whole herd hoof trimming, depending on herd size.

Comparing the net benefits for various herd sizes resulted in a linear increase in the difference in net benefits. The bigger the herd, the more cost-effective the partial herd trim became with an increase in the difference in net benefits of more than US$2,000 per increase at 50 cows (Fig 3).

#### Scenario 4

In the non-quota scenario with the direct cost of milk loss when having an SU, the result of the model was similar compared to having the quota system. Partial herd hoof trimming was also the most cost-effective strategy 100% of the time, with a difference in net benefits of US$4,004 (95% CI: US$2,407–5,412).

## Discussion

Within this Markov model, we investigated the difference in net benefits of two commonly practiced hoof trimming strategies in Alberta, Canada, with every 2 months partial herd hoof trimming and whole herd hoof trimming every 6 months, when treating SU [13]. Despite only being described in depth in Alberta, Canada, these two strategies are applied in entire Western Canada, according to the hoof trimmers of the Western Canadian Certified Hoof Trimmer’s Association (WCCHTA). Also, a Colombian study referred to these strategies reported by in Alberta [13, 43], possibly implying broader acceptance without related research reporting on strategies.

Based on the reference case results, partial herd hoof trimming was the cost-effective strategy over whole herd hoof trimming, with a difference in net benefits of US$4,337 (95% CI: US$2,713–5,830). In this model, we compared the costs of the hoof trimming strategies to the costs associated with the negative effects of SU on productivity, fertility, and increased labor. If all these parameters were included, early and targeted intervention decreased the costs of SU. If a cow affected by SU is trimmed in a partial herd hoof trimming, it is affected by SU for a shorter interval than it might be when waiting to be trimmed in a 6-month interval.

In the deterministic one-way sensitivity analysis, the probability of being trimmed was one of the sources of the highest uncertainty in the model. Therefore, we performed scenario analyses about the probability of being trimmed to elaborate on the impact of that variable on the cost-effective strategy. In Scenario 1, where the probability of getting trimmed in partial herd hoof trimming was 18% overall and also for cows with SU and cows without SU, whole herd hoof trimming was 98.6% of the time the most cost-effective strategy. A possible explanation is that when selecting cows randomly, those targeted for trims are not necessarily those affected by SU. Therefore, more cows would be affected by the negative impact of SU over a longer interval, which would increase costs. For whole herd hoof trimming, however, all cows get trimmed which would stop the negative effects of SU, even if the trim was not targeted. This reinforced that targeted intervention is the key to reducing SU-related costs.

In Scenario 2, we compared the reference case, where cows with SU had a 54% probability of being trimmed based on lameness status, to a 100% probability of getting trimmed for a cow with SU and an 18% probability of getting trimmed for a cow with SU. For all three options, the overall probability was 18%, which was described as the average percentage of a herd that gets trimmed in a partial herd trim in Alberta [13]. The difference in net benefits increased with the higher probability of trimming a cow with SU, and therefore partial herd hoof trimming became even more profitable. Also, from an animal welfare perspective, partial herd trim was the favorable strategy, because a cow presenting with lameness and potentially affected by SU would be treated in a timelier manner. Lameness in general has a negative impact on animal welfare, therefore all cows affected by lameness caused by hoof lesions would benefit from early intervention (hoof trimming) [44–46]. Also, with increasing herd sizes, partial herd trims might be more feasible. It would be interesting to further investigate whether hoof trimmers prefer partial or whole herd hoof trimming.

Another source of uncertainty in the sensitivity analysis was herd size. With the lower limit of 50 cows, the difference in net benefits was close to US$2,000, whereas at 500 cows, the difference in net benefits was close to US$23,000. Consequently, the bigger the herd, the greater the potential benefit of a partial herd hoof trim. With a fixed prevalence of 6.4%, in a small herd, fewer cows have an SU, whereas, in a big herd, more cows are affected not just by SU, but also by the negative effects of that lesion. For Scenario 3, this relationship was visually displayed and supported the theory of increased difference in net benefits depending on herd size. This emphasized the benefits of partial herd hoof trimming when performed targeted, especially as herd sizes are generally increasing.

Only costs for on-farm labor and decreased fertility, resulting in additional AI, were lower for whole herd versus partial herd hoof trimming. The lower labor costs were explained by the duration it takes to prepare cows for the hoof trimmer. Whereas it only takes 10 minutes to put all cows in one pen for the hoof trimmer in a whole herd hoof trimming, it takes 45 minutes to sort and select cows and put them in a separate pen for a partial herd hoof trimming, thereby accounting for the difference in on-farm labor costs.

Surprisingly, the costs for decreased fertility, accounted for in the model as two additional AIs needed (three versus five AIs for cows without versus with SU, respectively), were lower for whole herd hoof trimming. This might be explained by the gestation length of the cow and the impact on fertility only present in the first 3–5 months in milk till pregnancy is established; Inseminations are therefore an annual cost, however, in a partial herd hoof trim, a cow with a SU may not be trimmed and therefore be chronically affected by SU which is not accounted for in our model.

Hoof trimming, however, also has a preventive effect on hoof lesions, including SU [25]. In a Great Britain´s study producers were surveyed about undertaking preventive hoof trimming. The study results suggest that preventive hoof trimming was associated with increased herd yield [26]. However, no data was available to quantify the economic impact of prevention as expert opinion could not provide more clarity. With the preventive effect of hoof trimming, there may be an underestimation of net benefits, because due to the preventive effect, fewer cows will be affected by decreased milk production and decreased fertility due to SU. However, the preventive effect of hoof trimming is still a topic that needs further research but is beyond the scope of this current research.

Whole herd hoof trimming in this study assumed that 100% of the dairy cows of a herd get trimmed. However, on average 16% of the cows in a dairy herd are dry (60 days dry period and a 305-day lactation) and not every producer includes dry cows in hoof trimming. However, we decided to assume 100% of the herd in the Markov model for three reasons: 1) there is no literature/data available regarding whether these cows are excluded from trimming; 2) hoof trimmers reported that they regularly trim dry cows; and 3) the uncertainty around the model inputs is included with the probabilistic simulation.

In this model, it was assumed that the subset of 18% of cows trimmed during partial herd hoof trim [13], were selected based on whether they were lame or not, with 54% of cows with SU being lame and therefore identified for trimming [3]. In our experience, each producer optimizes their strategy to select cows for a partial herd hoof trim session. For example, cows are chosen due to the presence of lameness, or based on SU or other lesions during the previous trim. A hoof trimmer could support the producer in choosing cows for hoof trimming, or producers may use technology with lameness alert systems. Due to low sensitivity in proper identifying lameness by the producer [47, 48], this might result in potential overestimation. However, precision livestock farming could provide support for producers in monitoring lameness in the future and therefore contribute to early detection of lameness [46].

Animal welfare is of the utmost importance when discussing trimming strategies in hoof health management; therefore, it is strongly recommended and a moral obligation to treat lame cows immediately. However, in the Albertan study, investigating hoof trimming strategies [13], it is not mentioned whether farmers trimmed lame cows themselves in between hoof trimming visits by a hoof trimmer. Moreover, in another Albertan study, dairy farmers reported that lameness is hard to diagnose and often only severely lame cows are recognized and might therefore be addressed differently on farms [18]. One New Zealand study addresses the issue of the interval between detection of and treatment for lameness. When farm staff was performing locomotion scoring, only 75% of cows with a locomotion score of 3 were treated with more than 65% of treatments performed over 3 weeks later to the initial scoring result [17]. These studies emphasize the issue of cows receiving delayed treatment for lameness and increased awareness of not just the economic but also welfare implications are warranted. However, data on whether producers trim lame themselves between the hoof trimmer´s visits was not available for this study. Also, a 2023 study reports the issue that some data on lameness, needed for economic models were never reported in the literature and are therefore based on expert opinion as is the case for our models, and might have introduced biased estimations [49].

## Conclusion

In our model, partial herd hoof trimming was more cost-effective than whole herd hoof trimming, given the parameters used. However, the model implied that proper identification of lame cows to potentially include cows with SU may increase the difference in net benefits of partial versus whole herd hoof trimming, whereas when cows are chosen completely randomly for partial herd hoof trimming, whole herd hoof trimming is the more cost-effective strategy. In conclusion, the net benefit was dependent on targeted intervention with economic benefits related to the more frequent interventions. Targeted and early intervention also improves animal welfare with an SU present for a shorter period of time.
